# Effects of Enriched Environment on COX-2, Leptin and Eicosanoids in a Mouse Model of Breast Cancer

**DOI:** 10.1371/journal.pone.0051525

**Published:** 2012-12-13

**Authors:** Rachida Nachat-Kappes, Alexandre Pinel, Kristell Combe, Bruno Lamas, Marie-Chantal Farges, Adrien Rossary, Nicolas Goncalves-Mendes, Florence Caldefie-Chezet, Marie-Paule Vasson, Samar Basu

**Affiliations:** 1 Chaire d’Excellence Program, Biochemistry, Molecular Biology and Nutrition, Université d'Auvergne, Unité de Nutrition Humaine, Equipe ECRIN, CLARA, CRNH Auvergne; INRA, UMR, UNH, CRNH Auvergne, Clermont-Ferrand, France; 2 Centre de Lutte Contre le Cancer Jean Perrin, Unité de Nutrition, Clermont-Ferrand, France; 3 CHU Clermont-Ferrand, Service Nutrition, Clermont-Ferrand, France; 4 Oxidative Stress and Inflammation, Department of Public Health and Caring Sciences, Faculty of Medicine, Uppsala University, Uppsala, Sweden; University of North Carolina School of Medicine, United States of America

## Abstract

Cyclooxygenase-2 (COX-2) and adipokines have been implicated in breast cancer. This study investigated a possible link between COX-2 and adipokines in the development of mammary tumors. A model of environmental enrichment (EE), known to reduce tumor growth was used for a syngeneic murine model of mammary carcinoma. 3-week-old, female C57BL/6 mice were housed in standard environment (SE) or EE cages for 9 weeks and transplanted orthotopically with syngeneic EO771 adenocarcinoma cells into the right inguinal mammary fat pad. EE housing influenced mammary gland development with a decrease in COX-2 expressing cells and enhanced side-branching and advanced development of alveolar structures of the mammary gland. Tumor volume and weight were decreased in EE housed mice and were associated with a reduction in COX-2 and Ki67 levels, and an increase in caspase-3 levels. In tumors of SE mice, high COX-2 expression correlated with enhanced leptin detection. Non-tumor-bearing EE mice showed a significant increase in adiponectin levels but no change in those of leptin, F_2_-isoprostanes, PGF_2α_, IL-6, TNF-α, PAI-1, and MCP-1 levels. Both tumor-bearing groups (SE and EE housing) had increased resistin, IL-6, TNF-α, PAI-1 and MCP-1 levels irrespective of the different housing environment demonstrating higher inflammatory response due to the presence of the tumor. This study demonstrates that EE housing influenced normal mammary gland development and inhibited mammary tumor growth resulting in a marked decrease in intratumoral COX-2 activity and an increase in the plasma ratio of adiponectin/leptin levels.

## Introduction

Breast cancer is the second leading cause of deaths in women and is the most common cancer among women. Exposure to high levels of stress, defined as an alteration of the body’s hormonal and neuronal secretions caused by the central nervous system in response to a perceived threat, may have an effect on mammary gland development and affect breast cancer risk [1]. In particular, traumatic events in childhood or adolescence were found to be significantly associated with increased risk of breast cancer [1], [2]. Housing rodents in an enriched environment (EE) is a classic model that has been extensively utilized for studying the effect of a combination of complex inanimate and social stimulations. It consists of a complex housing with increased space for exploration filled with a variety of inanimate objects that enhances sensory, cognitive, motor and social stimulation relative to standard housing conditions [3].

EE is considered as a positive stress or “eustress”, and one of its most important effects of EE is a reduction in the anxiety levels of the residents. In addition, it has been shown to induce many beneficial effects, such as improved cognition, recovery from nervous system injuries and disorders, accelerated development of the visual system and even decreased the vulnerability to various addictions on rodents [3], [4], [5], [6], [7]. In addition, EE significantly reduces cancer burden syngeneic melanoma and colon cancer models [8]. Socially isolated female T-antigen transgenic mice (C3(1)/SV40) in contrast develop larger mammary gland tumors than group-housed mice [9], [10]. However, certain effects of EE have been reported as sex-specific in C57BL/6J mice: four weeks of EE during adolescence phase of development had an anxiolytic effect in male mice but an anxiogenic effect in females [11].

Adipokines and proinflammatory mediators, particularly cyclooxygenase-2 (COX-2) are two major factors related to mammary cancer progression in addition to the well-known role of free radicals in cancer initiation [12], [13]. Thus, eicosanoids, in particular the bioactive prostaglandins and isoprostanes formed through COX and free radicals, respectively may play a part in breast cancer [14]. We speculated that all these factors may have varying effects depending on whether mice are housed in EE or standard environment (SE). To assess the effects of EE on mammary gland and cancer development and their impact on adipokines and inflammation, we used an orthotopic model, in which EO771, a mammary gland medullary adenocarcinoma cell line, is injected into the mammary fat pads of immune-competent C57BL/6 mice. The EO771 cells were derived from the mammary tumors of C57BL/6 female mice that were estrogen receptor-positive [15], [16], [17]. When transplanted subcutaneously near the fat pad of the fourth mammary gland in the lower abdomen of syngeneic mice, EO771 cells grow into solid tumors and can eventually invade other organs such as the intestinal mesentery, pancreas, diaphragm, peritoneal wall and the lung [17].

The aim of this study was to investigate a possible link between COX-2 and adipokines in the development of mammary tumors. To determine this, environmental enrichment (EE), known to reduce tumor growth was used in a syngeneic murine model of mammary carcinoma. We have found that EE housing influenced normal mammary gland development and inhibited mammary tumor growth resulting in a marked decrease in intratumoral COX-2 activity and an increase in the plasma ratio of adiponectin/leptin levels.

## Results

### Effects of Housing on Body Weight, Body Composition and Organs Weight

**Figure 1:**
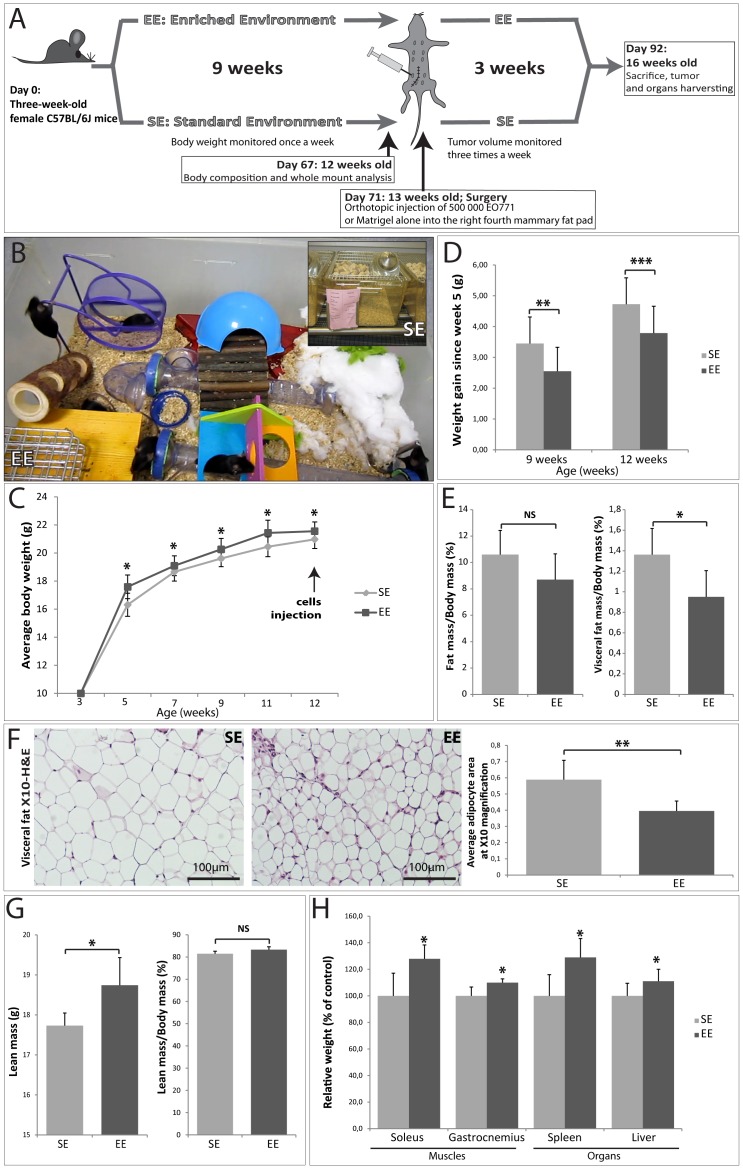
Effect of 9 weeks of SE and EE housing on body weight and body composition. (A) Schematic diagram illustrating the experimental protocol. (B) EE and SE cages (see additional supplementation video 1). (C) Mean body weight growth per housing condition (n = 25 per group). *Ad-libitum–fed mice* were housed in the EE or SE cages for 9 weeks prior to tumor injection. (D) Levels of weight gained by 9 and 12-week-old EE and SE mice since the fifth postnatal week (n = 25 per group). (E) Left panel: Analysis of total fat mass by magnetic resonance spectroscopy and normalized to total body mass of 12-week-old mice. Right panel: Analysis of excised visceral fat mass normalized to total body mass of 12-week-old (n = 6 per group). (F) Left panel: Representative hematoxylin and eosin-stained visceral fat sections from 12-week-old EE and SE mice. Right panel: Adipocyte area was measured using images of visceral fat sections and ImageJ software (n = 3 per group). (G) Left panel: Measurement of lean mass by magnetic resonance spectroscopy of 12-week-old mice (n = 7 per group). Right panel: Measurement of percent of lean mass by magnetic resonance spectroscopy, normalized to total body mass of 12-week-old (n = 7 per group). (H) Relative weight of soleus, gastrocnemius, spleen and liver of 12-week-old (n = 7). The SE levels were set at 100%. Data presented are the mean ± SEM; * = p<0.05, ** = p<0.01, *** = p<0.001, NS = not significant.

The body weight of mice housed in EE or SE cages were measured every other week for a period of 9 weeks after weaning and 2 weeks of adaptation (from 5 weeks to 12 weeks of age). Mice housed in EE weighed more than their counterparts housed in SE cages after 2 weeks of adaptation (5 weeks of age) (Figure 1C). After 9 weeks of EE or SE housing (12 weeks of age), EE mice exhibited a slight but significant increase in body weight up to 5%, as compared to SE mice (Figure 1C). However, after 4 weeks (from 5 weeks to 9 weeks of age) EE mice gained almost one gram less body weight than SE mice (2.6±0.8 vs. 3.5±0.9 g; Figure 1D). After 7 weeks (from 5 weeks to 12 weeks of age) EE mice gained less than 4 g of body weight, whereas SE mice gained nearly 5 g (3.7±0.8 vs. 4.8±0.8 g; Figure 1D). Body composition assessed in ∼12-week showed no significant decrease in total fat mass in EE mice (Figure 1E, left panel). However, EE housing significantly decreased the visceral fat mass up to 29% (Figure 1E, right panel). In addition, to determine if the decreased mass in EE mice could be secondary to reduced visceral adipocyte size, we performed H&E staining on the excised visceral fat of EE and SE mice (Figure 1F). The adipocytes visualized in the visceral fat tissue of EE mice were about 32% fewer than those of SE mice (Figure 1F). EE mice had about 5% more lean mass than SE mice (18.7±0.7 vs. 17.8±0.3 g; Figure 1G, left panel). However, there were no significant differences were noticed in lean mass after adjusting for total body mass (Figure 1G, right panel). EE mice had a significant increase in gastrocnemius (+10%; 219±6.33 vs. 199±13.2 mg) and soleus (+27.6%; 16.2±2.2 vs. 12.7±1.7 mg) muscles weight compared to SE mice (Figure 1H) but, no significance difference was observed in tibialis anterior, plantaris or EDL muscles (data not shown). The spleen and liver of EE mice were significantly more enlarged than those of SE mice (+29%; 103±14.6 vs. 80±12.7 mg and +11%, 864±74.2 vs 778±77.7 mg respectively) (Figure 1H). In addition, while we were monitoring mice weight from 5 to 12 weeks old, we observed significant barbering activity in mice in SE cages with behaviour-associated hair loss and whisker but none at all in mice housed in EE (data not shown). These observations demonstrate that EE induces a mild and positive stress compared to SE housing [18].

### Effects of Housing on Mammary Gland Development

**Figure 2:**
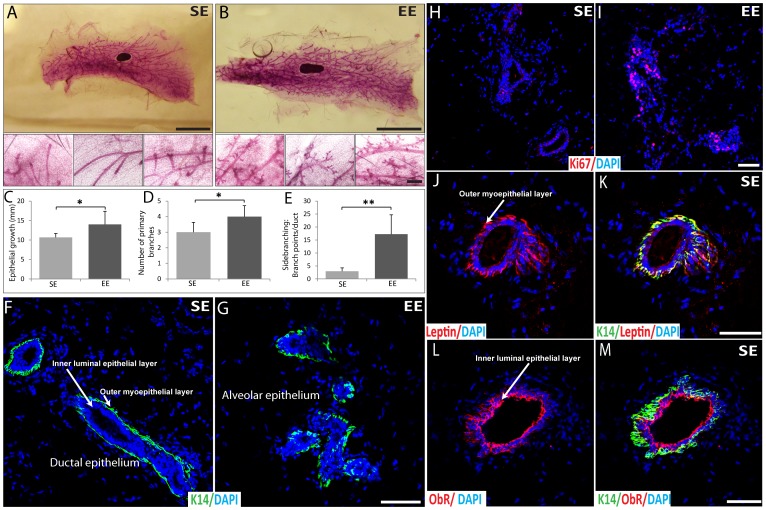
Analysis of normal mammary gland of 12-week-old mice housed in SE and EE cages for 9 weeks. (A, B) Carmine whole mount staining of the fourth mammary gland isolated from EE and SE mice. Lower panels show higher magnification of upper panels. (n = 6; scale bars; upper panels: 5 mm, lower panels: 250 µm). (C) Quantification of epithelial growth that corresponds to the distance from the lymph node to the end of epithelial tree, measured using a ruler in millimeters (mm) (n = 6). (D) Quantification of primary branches were defined as ducts extending from the nipple and terminating in an end bud (n = 6). (E) Quantification of the side-branching that corresponds to the number of branch points along the terminal ductal tips (n = 6). (F) Ductal epithelium observed in the mammary gland isolated from SE mice labeled by indirect immunofluorescence for keratin 14 (K14, green) and with DAPI as nuclear counterstain (blue). (G) Alveolar epithelium observed in the mammary gland isolated from EE mice labeled by indirect immunofluorescence for keratin 14 (K14, green) and with DAPI as nuclear counterstain (blue). (H–I) Normal mammary gland isolated from EE and SE mice labeled by indirect immunofluorescence for Ki-67 (red) and with DAPI as nuclear counterstain (blue). (J–K) Normal mammary gland isolated from SE mice labeled by indirect immunofluorescence for leptin (red), keratin 14 (K14, green) and DAPI (blue). (J) The staining pattern of leptin is showed in red. (K) The three color overlays are shown. (L–M) Normal mammary gland isolated from SE mice labeled by indirect immunofluorescence for leptin receptor (Ob-R, red), K14 (green) and DAPI (blue). (L) The staining pattern of leptin receptor is showed in red. (M) The three color overlays are shown. Scale bars: 50 µm. Data presented are the mean ± SEM; * = p<0.05, ** = p<0.01.

Before the orthotopic transplantation of the mammary cell line, we analyzed the effects of EE and SE housing on mammary gland development in adult virgin mice aged 12 weeks, the time at which puberty ends and terminal end buds (TEBs) reach the edge of the fat pad and disappear (9 weeks in EE or SE cages). Surprisingly, whole-mounts of the fourth inguinal mammary gland revealed significant structural differences. There was extensive side-branching in EE animals, in contrast with the smooth surface seen in the ducts of SE mice (Figures 2A, 2B and 2E). We also observed increased epithelial growth and significantly more primary branches in the mammary fat pad of EE animals (Figure 2C and 2D). The mammary gland contains both ductal and alveolar epithelia [19], [20]. Ductal epithelium was evident as a single epithelial cell layer (Figure 2F), and alveolar epithelium was evident as clusters of small lobuloalveolar structures (Figure 2G). The inner layer of luminal epithelial cells present in both ductal and alveolar epithelia, is surrounded by a layer of myoepithelial cells expressing keratin 14 (K14) (Figure 2F, 2G, 2K and 2M). Alveolar epithelium was frequently observed in the mammary fat pad of EE mice (Figure 2G), while we only observed ductal epithelium in the mammary gland of SE mice (Figure 2F). To determine whether the enhanced side-branching and presence of alveolar epithelium of the mammary glands in EE mice is due to an increase in cell proliferation, we performed immunohistochemistry staining for Ki67 as a cell proliferative marker (Figure 2H and 2I). As expected, the extensive ductal side-branching observed in EE mice was coincident with a marked increase in cellular proliferation (Figure 2I). Since leptin acts as an autocrine/paracrine factor, which is involved in mammary gland development [21], we next analyzed the expression and localization of leptin and its receptor (ObR) in the normal mammary gland of 12-week-old adult virgin SE and EE mice (Figure 2J, 2K, 2L and 2M). Leptin was strongly detected in the cytoplasm of the outer myoepithelial cells in the mammary ducts of SE mice (Figure 2J), where it co-localized with keratin 14 (K14) (Figure 2K, indicated in yellow). ObR was detected in the inner luminal epithelial layer of the mammary ducts at the cell-cell borders (Figure 2L and 2M). Both leptin and ObR antibodies exhibited similar staining patterns and intensities in the mammary ducts of EE mice (data not shown).

### Effects of Housing on Mammary Tumor Growth

**Figure 3:**
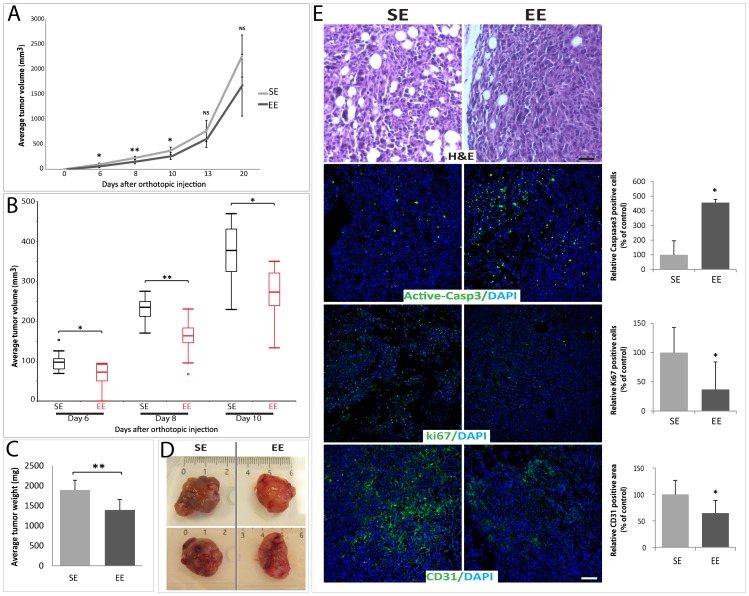
Mammary tumor growth in EE and SE housing. (A) Growth of tumor volume in SE and EE mice, EE decreased tumor growth rate (n = 10–12 per group). (B) Significant decrease of tumor volume observed at day 6, 8 and 10 shown in a box and whisker plot showing median, quartiles, and extreme values (outliers are shown as circles) (n = 10–12 per group). (C) 21 days after the injection, the tumors were excised from SE and EE mice and weighted (n = 8 in each group). (D) Representative EO771 adenocarcinoma dissected day 21 after orthotopic injection into the right fourth mammary fat pad. (E) Upper panels: Representative hematoxylin and eosin-stained tumor sections excised from EE and SE mice, 21 days after the injection. Lower panels: Tumor sections were labeled by indirect immunofluorescence staining for active caspase 3, for Ki67 or for CD31 (green) and with DAPI as nuclear counterstain (blue). Right panel: The immunolabeled cells for active caspase 3 and Ki67 and the CD31 positive area was measured using ImageJ software (n = 3 per group). The SE levels were set at 100%. Scale bars: 100 µm. Data presented are the mean ± SEM; * = p<0.05, ** = p<0.01, NS = not significant.

Tumor volume was monitored with a caliper three times a week (Figure 3A). In the mice housed in EE for 9 weeks prior to tumor implantation, the mean volumes of the mammary tumors were significantly reduced at days 6, 8 and 10 after the injection (−34%; −31% and –29%, respectively) (Figure 3A and 3B). Six days after the injection, all SE mice had developed solid tumors, whereas in 7% of EE mice had no palpable tumors were found (Figure 3B). However, 8 days after the transplantation all EE mice exibited palpable tumors, demonstrating that the occurrence of visible tumors can be delayed. From day 13 after the inoculation, EE mice still had smaller tumors than those in SE mice but no longer to a level of significance owing to the high variation in the induced-tumor volume in both groups (Figure 3A). As with the volume, the weight of the tumor determined at sacrifice was significantly lower (−26%) in the EE mice (Figure 3C). EO771 cells formed solid and highly hemorrhagic tumors (Figure 3D). The macroscopic appearance of representative tumors excised at the time of sacrifice suggested that tumors in EE mice were less hemorrhagic than those in SE mice (Figure 3D). At the time of sacrifice, the lungs and other organs were analyzed for the development of distant metastasis. None was observed in EE and SE tumor-bearing mice (data not shown). Hematoxylin and eosin staining of the tumor showed that the border of the tumor with the normal mammary gland was infiltrated by numerous lipid vacuoles in both EE and SE mice (Figure 3E, upper panel). However, only very rare or no lipid vacuole infiltrations were observed in the deep portion of the tumor (data not shown). Reduced tumor size in EE mice was associated with an increase in apoptosis and a decrease in proliferation as shown by active caspase 3 and Ki67 immunostainings, respectively (Figure 3E, second and third panels). Since tumor isolated from SE mice looked more hemorrhagic than those from EE mice (Figure 3D), we analyzed tumor angiogenesis, with cluster of differentiation 31 (CD31) immunostaining. As expected, tumors in SE mice had a significantly elevated relative microvessel area, compared to the tumors in EE mice (Figure 3E, lower panel).

### Effects of Housing on COX-2 and Leptin Expression in Normal Mammary Gland

**Figure 4:**
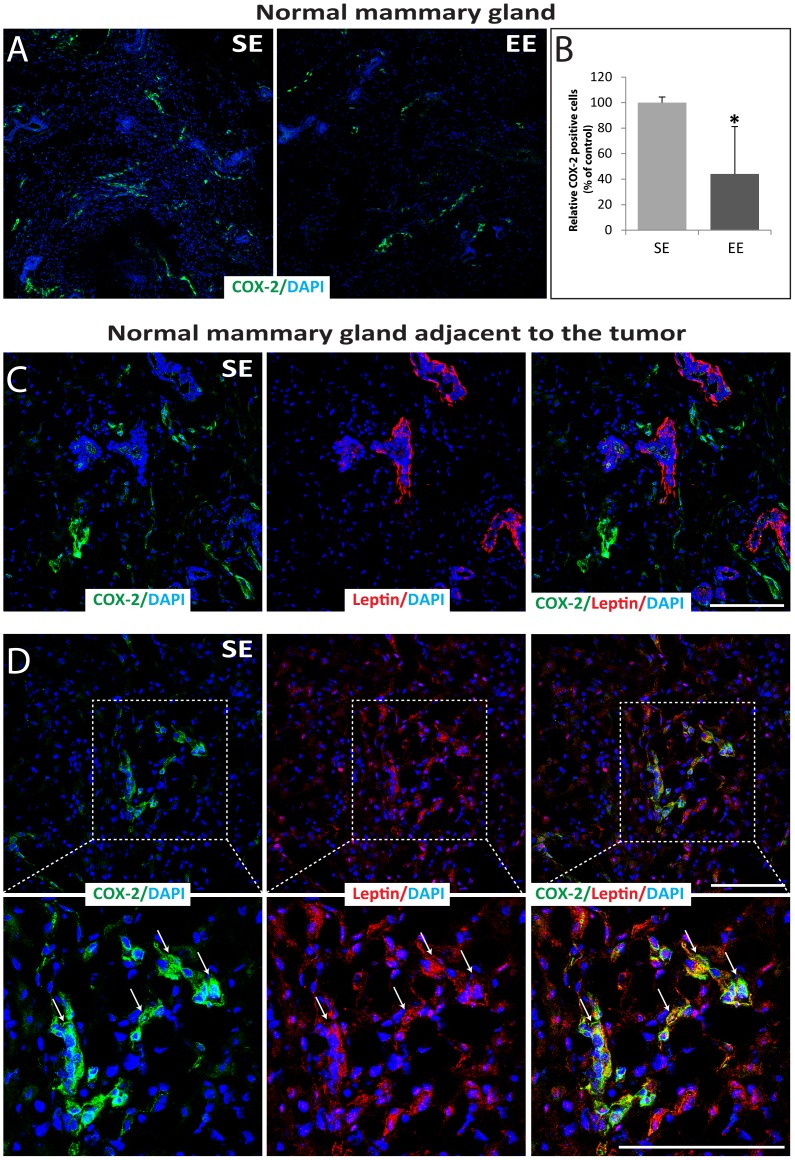
COX-2 expression in normal mammary gland from 12-week-old mice housed for 9 weeks in SE compare to EE. (A) Normal mammary gland sections were labeled by indirect immunofluorescence staining for COX-2 (green) and with DAPI as nuclear counterstain (blue). (B) Immunolabeled cells for COX-2 in the normal mammary gland were quantified using ImageJ software where the SE levels were set at 100% (n = 3 per group). (C–D) Normal mammary gland adjacent to a tumor excised from a SE mouse was cut and labeled by indirect immunofluorescence staining for COX-2 (green), leptin (red) and with DAPI as nuclear counterstain (blue). Right panels show overlays of the left and middle panels. In overlay images, the yellow shows co-localization of COX-2 and leptin. Lower panels show boxed regions at high magnification. Scale bars: 100 µm. Data presented are the mean ± SEM; * = p<0.05, NS = not significant.

Firstly, the COX-2 expression was assessed by indirect immunofluorescence staining and confocal microscopy analysis in normal healthy mammary glands. COX-2 was detected in stromal-vascular and adipocyte fractions in normal mammary glands of SE and EE mice without any injection (Figure 4A). In addition, the mean score of COX-2 positive cells was higher in SE mice than in EE mice (Figure 4B). Secondly, in normal tissue adjacent to the tumors of SE mice, we observed a very strong leptin staining in the basal layer of myoepithelial cells of the mammary ducts (Figure 4C) as above-mentioned in normal healthy mammary gland (Figure 2J). COX-2 was also detected in the stromal-vascular and/or adipocyte fractions of the mammary gland where it partially co-localized within cells expressing high levels of leptin (Figure 4D, white arrows). We made similar observations in normal mammary gland adjacent to the tumors of EE mice (data not shown).

### Effects of Housing on COX-2, Leptin and Eicosanoids Expression in Tumors

**Figure 5:**
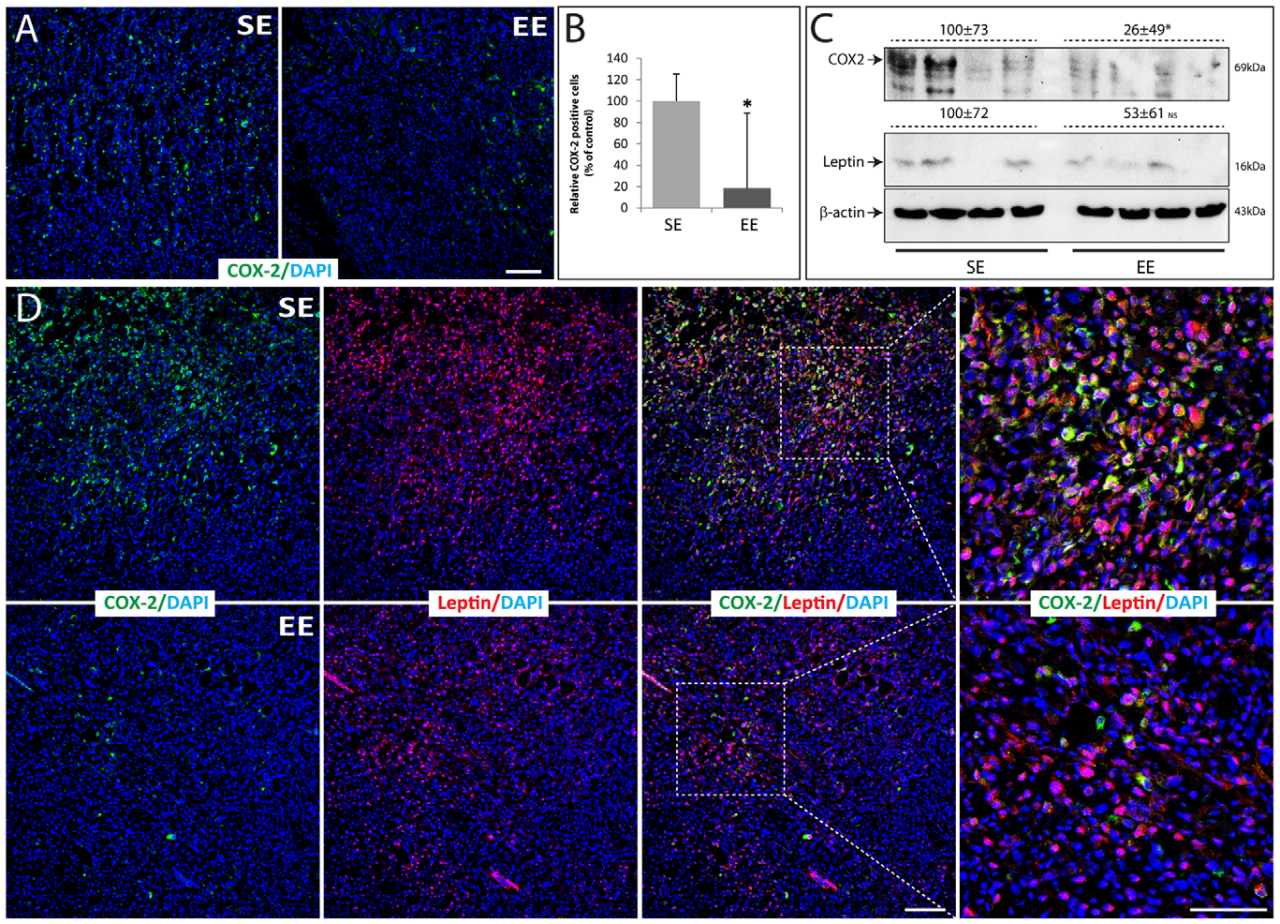
COX-2 expression in tumors from 12-week-old mice housed for 9 weeks in SE compare to EE. (A) Tumor excised from EE and SE mice, 21 days after the injection were cut and labeled by indirect immunofluorescence staining for COX-2 (green) and with DAPI as nuclear counterstain (blue). (B) Immunolabeled cells for COX-2 into the tumors were quantified using ImageJ software where the SE levels were set at 100% (n = 3 per group). (C) Tumor lysates were analyzed with western-blotting with the indicated antibodies. Photographs of chemiluminescent detection of the blot are shown. The relative abundance of each band to its own β-actin was quantified using ImageJ software, and the SE levels were set at 100%. (D) Tumors excised from SE and EE mice were cut and labeled by indirect immunofluorescence staining for COX-2 (green), leptin (red) and with DAPI as nuclear counterstain (blue). Right panels show overlays of the left and middle panels where the yellow shows co-localization of COX-2 and leptin. Right panels show boxed regions at high magnification. Scale bars: 100 µm. Data presented are the mean ± SEM; * = p<0.05, NS = not significant.

COX-2 expression and localization were determined in the tumors by indirect immunofluorescence analyses. Tumors from both groups showed COX-2 expression (Figure 5A). However, the mean score of COX-2 positive cells was higher in tumors of SE mice compared to those of EE mice (Figure 5B). Although great variability in COX-2 expression was observed, Western-blotting analysis of tumor cell lysates confirmed stronger COX-2 protein expression in tumor isolated from SE mice group than in tumors of EE mice (Figure 5C). Leptin expression levels in tumor extracts didn’t show any significant differences between SE and EE mice (Figure 5C). Interestingly, tumors isolated from SE mice with high COX-2 expression displayed detectable and high leptin expression (Figure 5C). Double immunostainings by indirect immunofluorescence for leptin and COX-2 in tumors, showed that leptin immunoreactivity was strongly detected in tumors in high COX-2-expressing areas, suggesting that a correlation exists between COX-2 and leptin expression (Figure 5D).

The presence of prostaglandin F_2α_ (PGF_2α_) and 8-iso-PGF_2α_, the eicosanoids formed by COX- and free radicals-catalysation of arachidonic acid, respectively, in the tumors was assessed by indirect immunofluorescence analyses (Figure S1). Both 8-iso-PGF_2α_ and PGF_2α_ metabolite (15-keto-dihydro-PGF_2α_) were detected in tumors from SE and EE mice but with a very high variability within the same tumor (Figure S1). Thus, no significant variations were achieved in 8-iso-PGF_2α_ and PGF_2α_ expression in the tumors between groups.

### Effects of Housing on Adipokines

**Figure 6:**
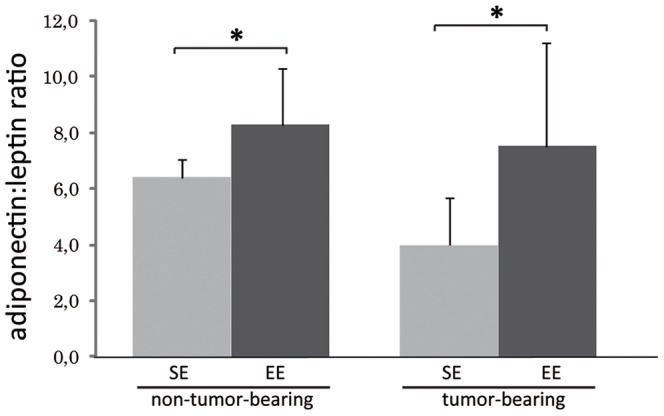
Analysis of adiponectin:leptin ratio in non-tumor-bearing and tumor-bearing EE and SE mice. Adiponectin:leptin ratio is significantly increased in non-tumor-bearing and tumor-bearing EE mice compared to SE mice.

Adiponectin levels were significantly increased by about 27% (6.99±1.38 vs. 8.85±2.24 µg/ml), in the plasma of EE non-tumor-bearing mice compared to SE non-tumor-bearing mice (Table 1). Interestingly, EE and SE tumor-bearing mice, showed a significant decrease in plasma adiponectin levels compared to EE and SE non-tumor-bearing mice which received vehicle only (−43% in both groups; Table 1), (6.99±1.38 vs. 4.06±1.18 µg/ml) and (8.85±2.24 vs. 4.97±1.66 µg/ml). No significant differences were observed in the plasma levels of leptin, resistin in SE and EE non-tumor-bearing mice. However, SE and EE tumor-bearing mice showed significant increases in resistin compared to those in SE and EE non-tumor-bearing mice. Remarkably, leptin levels were significantly decreased in EE tumor-bearing mice compared to those in EE non-tumor-bearing mice (0.46±0.28 vs. 1.07±0.59 ng/ml), and non-significantly decreased (p = 0.0568) in EE tumor-bearing mice compared to those in SE tumor-bearing mice (0.46±0.28 vs. 0.87±0.26 pg/ml) (Table 1). Figure 6 shows that the adiponectin:leptin ratio is significantly increased in EE non-tumor and tumor-bearing mice compared to those in SE non-tumor and tumor-bearing mice, respectively.

**Table 1:** Effect of SE and EE housing on the levels of various adipokines in plasma of C57BL/6 mice injected with vehicle (no tumor cells) or with EO771 cells (tumor).

	SE, no tumor (n = 7)	EE, no tumor (n = 7)	SE tumor	EE tumor	Two-way
			(n = 9)	(n = 9)	ANOVA
**Adiponectin (µg/ml)**	6.66±1.11*^a^*	8.18±1.26*^b^*	4.06±1.18*^c^*	5.24±1.61*^c^* *	e (p = 0.0441) t (p = 0.0005)
**Leptin (ng/ml)**	1.07±0.41*^a^*	1.34±0.57*^a^*	1.07±0.46*^a.b^*	0.94±0.51*^b^*	−
**Adiponectin/Leptin** **ratio**	7.08±3.02*^a^*	7.34±3.53*^a^*	4.47±2.21*^a^*	6.57±2.52*^a^*	−
**Resistin (ng/ml)**	2.69±0.83*^a^*	2.92±0.31*^a^*	3.27±0.74*^a.b^*	3.80±0.65*^b^*	−

*Different superscripts* denote significant difference at p<0.05 using the Mann-Whitney U test. A two-way multivariate analysis of variance was conducted to explore specific effects of the environment (e), the tumor (t) and the interaction between these two factors. Results are mean ± SEM.

* p = 0.0516 EE tumor *vs* SE tumor.

**Figure 7:**
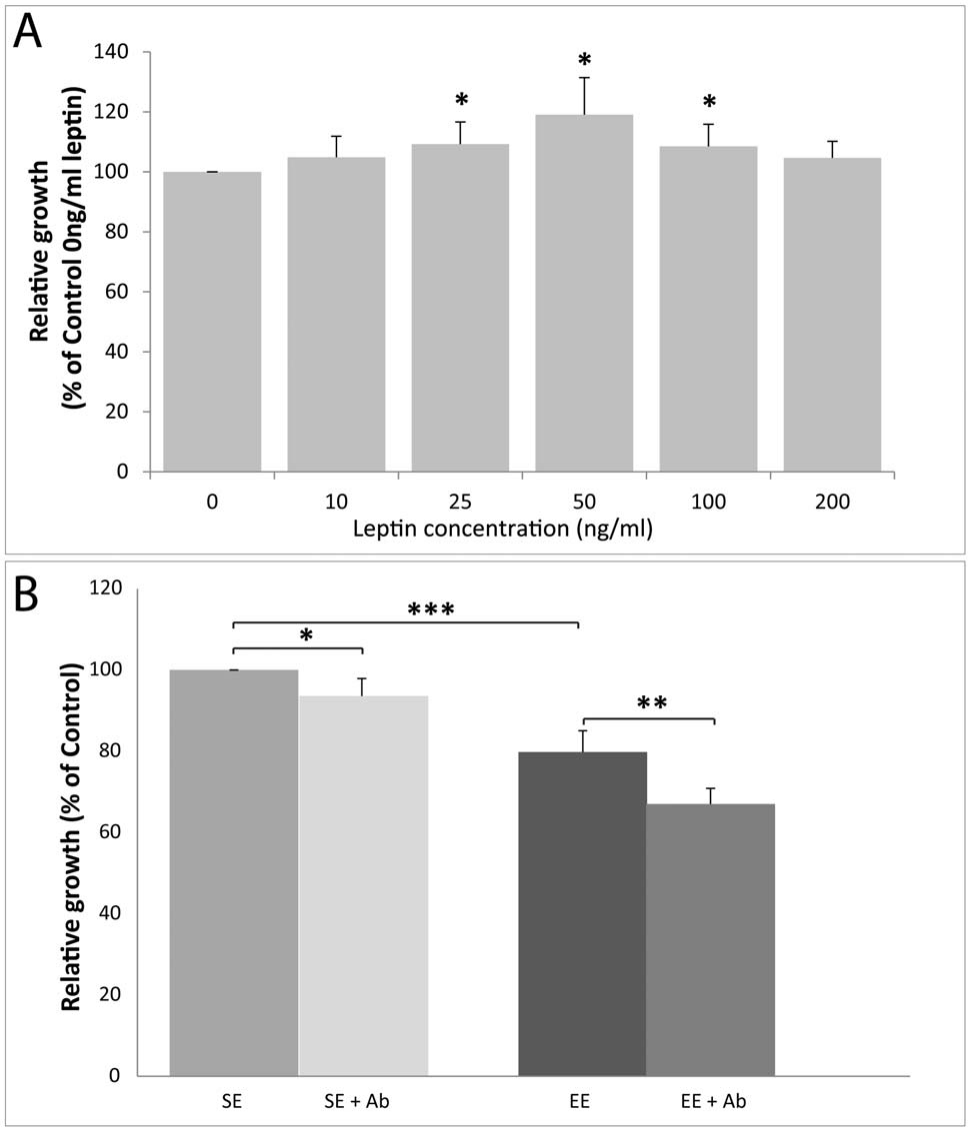
Effets of leptin and serum from 12-week-old mice housed in EE and SE for 9 weeks on EO771 cell proliferation. (A) EO771cells proliferation in response to leptin treatment at 0, 10, 25, 50, 100 and 200 ng/ml for 24 h (n = 3). Control levels (0 ng/ml) were set to 100%. (B) EO771 cells were cultured for 24 h with 1% of serum alone from 12-week-old mice housed in EE or SE cages for 9 weeks, or with 1% of serum pretreated with leptin-neutralizing antibody (n = 6 per group). Control levels (serum from SE mice) were set to 100%. Data presented are the mean ± SEM; * = p<0.05, ** = p<0.01, NS = not significant.

To determine whether leptin has a direct role in cell proliferation, E0771 cultured cells were incubated with increasing concentrations of leptin (0–200 ng/ml) prior to resazurin-based cell proliferation assay. Analyses of the conversion of resazurin to resorufin revealed an inverted U shape of the dose-response relationship between leptin and cell proliferation. The greatest effect of leptin was observed with the dose of 50 ng/ml (119±12% relative to vehicle control) (Figure 7A). As the serum levels of factors associated with survival and proliferation of cancer cells were influenced by EE, we next investigated whether EO771 adenocarcinoma cells incubated with serum from either EE or SE mice would impact on the cell growth *in vitro* using resazurin-based cell proliferation assay. Serum from EE mice significantly slowed the EO771 cells growth compared to SE mice serum. We further examined the direct role of leptin in tumor cell proliferation using a leptin-neutralizing antibody. Pretreatment of serum with leptin-neutralizing antibody significantly inhibited tumor cell growth (Figure 7B).

### Effects of Housing on Systemic Oxidative Stress and Inflammatory Markers

To evaluate systemic inflammation and oxidative stress, we measured the levels of 15-keto-dihydro-PGF_2α_ and 8-iso-PGF_2α_ in the urine of EE and SE tumor-bearing and non-tumor-bearing control mice. There were no significant changes in 8-iso-PGF_2α_ or 15-keto-dihydro-PGF_2α_ between SE and EE tumor-bearing or non-tumor-bearing mice (Table 2). No significant differences were observed in plasma levels of interleukin-6 (IL-6), tumor necrocis factor-α (TNF-α), monocyte chemoattractant protein-1 (MCP-1) and plasminogen activator inhibitor-1 (PAI-1) in SE and EE non-tumor-bearing mice. However, SE and EE tumor-bearing mice showed significant increases in IL-6, TNF-α, MCP-1 and PAI-1 compared to SE and EE non-tumor-bearing mice.

**Table 2:** Effect of SE and EE housing on the levels of PGF_2α_ metabolite and F_2_-isoprostane in the urine, and inflammatory markers in plasma of C57BL/6 mice injected with vehicle (no tumor cells) or with EO771 cells (tumor).

	SE, no tumor (n = 7)	EE, no tumor (n = 7)	SE tumor (n = 9)	EE tumor (n = 9)
**8-Iso-PGF_2α_, nmol/** **mmol creatinine**	1.86±0.39	1.87±0.30	1.82±0.59	2.03±0.59
**PGF_2α_ metabolite,** **nmol/mmol** **creatinine**	0.77±0.25	0.80±0.16	0.87±0.57	0.66±0.17
**TNF-α** **(pg/ml)**	2.35±1.64*^a^*	2.21±0.96*^a^*	10.07±2.78*^b^*	10.58±2.20*^b^*
**IL-6** **(pg/ml)**	3.65±1.57*^a^*	5.63±2.75*^a^*	22.83±11.81*^b^*	21.90±10.47*^b^*
**MCP-1** **(pg/ml)**	18.29±9.40*^a^*	17.89±9.63*^a^*	242.4±107.5*^b^*	164.2±91.6*^b^*
**PAI-1** **(ng/ml)**	0.48±0.16*^a^*	0.84±0.41*^a^*	3.26±0.64*^b^*	3.06±1.23*^b^*

Mice were subjected to SE or EE housing for 9 weeks and injected with EO771 cells or vehicle only as described in the “Materials and Methods”. At 21 days after the EO771 cells injection, urine and blood samples were collected from the mice. Plasma levels of adiponectin were measured by ELISA and interleukin (IL)-6, leptin, tumor necrosis factor (TNF)-α, resistin, plasminogen activator inhibitor-1 (PAI-1) and monocyte chemoattractant protein-1 (MCP-1) were measured with Luminex multiplexed assay. 15-Keto-dihydro-PGF_2α_ and 8-iso-PGF_2α_ were measured in urine by radioimmunoassay. *Different superscripts* denote significant difference at p<0.05. Results are mean ± SEM.

## Discussion

This study was designed to assess whether physical and social environmental enrichment induces differential effects on tumor growth and, and if so, to determine their relation to oxidative stress, inflammatory and metabolic consequences using a mouse model of breast cancer. We show for the first time that EE housing influences mammary gland development. Additionally, we observed that normal mammary glands of mice housed in SE conditions have more COX-2 positive cells than those of mice housed in EE condition. These findings suggest an increased inflammatory state of the mammary gland in SE mice as observed previously with obese mice [22]. In addition, the significantly reduced mammary tumor growth observed in EE mice was related to lower COX-2 expression while a higher COX-2 expression together with increased leptin detection was observed in tumors that had developed in SE mice.

EE has known to have an impact on behavioral and physiological mechanisms in rodents in a sex-specific manner [11], [23], [24]. Indeed, EE decreases body weight of male C57BL/6 mice [8], while we observed a significant increase in the body weight of female mice. However, the slower weight gain observed in EE mice from week 5 to weeks 9 and 12 may have been in part be due to the reduced visceral fat accumulation. Animals kept in EE housing have been shown to decrease adiposity and to induce a switch from transforming white adipose tissue (WAT) into brown adipose tissue (BAT) [25]. WAT and BAT perform very different functions in mammals since WAT stores surplus of energy mainly in the form of as triacylglycerols while BAT dissipates energy directly as heat [26]. This switch from WAT to BAT could explain the reduction in visceral fat mass in EE mice.

**Figure 8:**
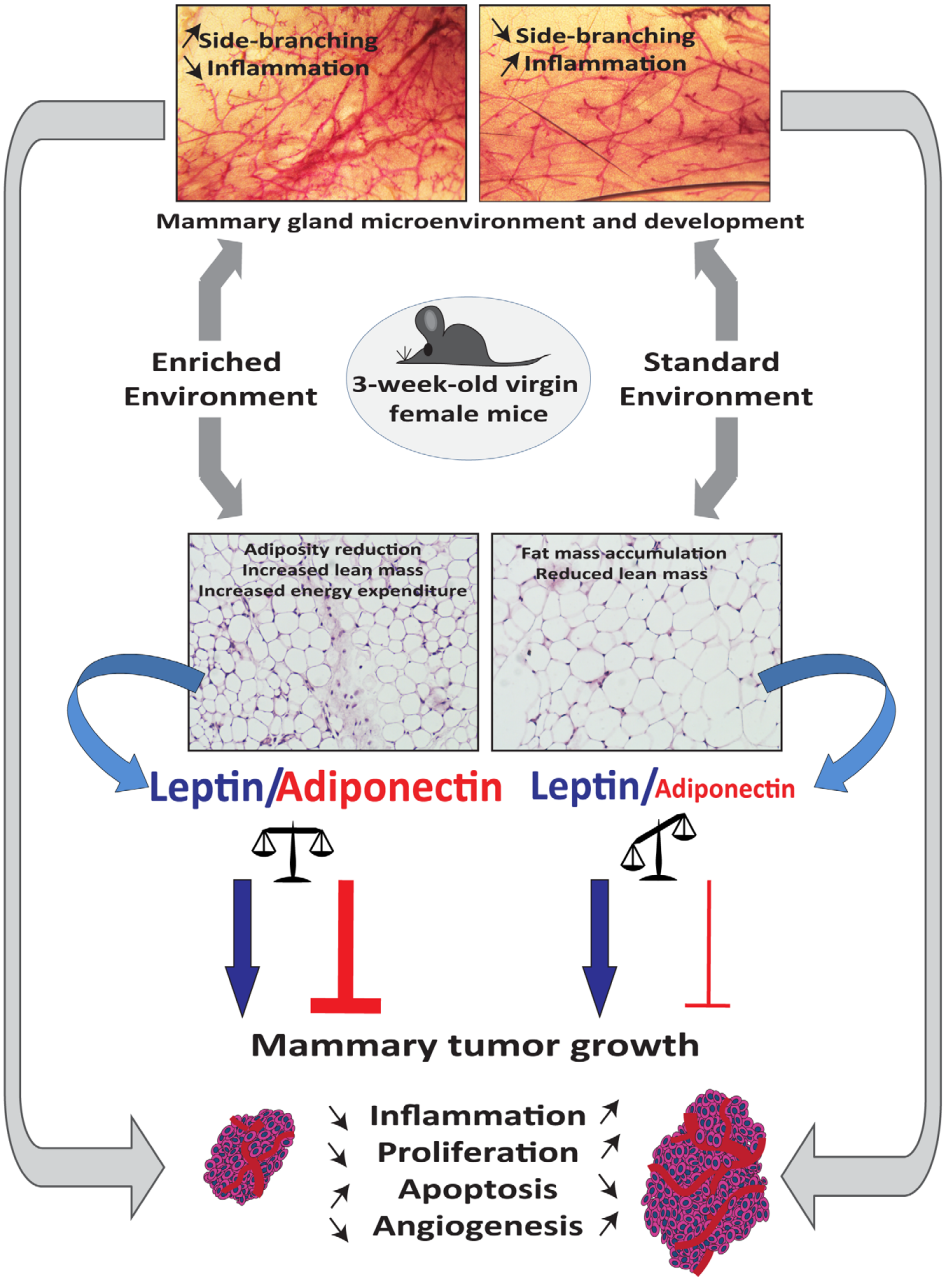
Effects of EE housing from postweaning period to adulthood on mammary gland development and mammary tumor growth. Environmental enrichment starting from postweaning to 9 weeks results in increase mammary ductal complexity in 12 week-old virgin adult mice compared to mice housed in standard laboratory conditions. Thus, the important changes in mammary gland development suggest that EE housing affects mammary microenvironment. Moreover, the mammary microenvironment is now recognized as a key participant in tumor progression. EE housing leads to a significant decrease in mammary tumor growth. Reduction of mammary tumor growth is associated with a decrease in cell proliferation, inflammation and angiogenesis, and with increased apoptosis. Balance between serum adiponectin and leptin, is a critical factor in mammary tumorigenesis. The adiponectin to leptin ratio is higher in EE housed mice compared to SE housed mice, and may contribute to the inhibition of tumor growth. Finally, SE housing is associated with elevated inflammation in the mouse mammary gland and can potentially drive tumor progression.

This study reports the effects of SE and EE housing on mammary gland development in mice. The growth of most cancers, including breast cancer, is partly dependent on the local tissue microenvironment. Thus, we first analyzed the effects of housing on the adult virgin mouse mammary gland, before transplantation of cell line. Intriguingly, we observed specific notable changes in mammary gland morphology demonstrating that unlike housing environment influences differently mammary gland development, and thus, the mammary microenvironment (Figure 8). Several different animal models have shown similar morphologic changes during mammary gland development [27], [28], [29]. Mammary gland observed in our SE mice tend to look like mammary glands of mice subjected to high-fat diet with less frequent side-branching [29]. Moreover, it has been previously shown that neonatal maternal separation, over the first 3 weeks of life, causes precocious mammary gland development and induces a higher incidence and shorter latency of carcinogen-induced mammary tumors in female BALB/c mice subjected to long but not short separation [27]. When stress induced by neonatal maternal separation, increased ductal side-branching was observed in EE mammary glands. In addition, at 14 weeks of age, isolate-housed female FVB/N mice just after weaning, showed strikingly reduced mammary gland development compared to animals housed in standard conditions, as observed in our SE mice housed in standard laboratory cages [28]. These different models, including EE housing, demonstrate that mammary gland development is strongly influenced either by diet [29], [30] or by exposure to stress in early life and during adolescence [28]. Since both the hypothalamic-pituitary-adrenal (HPA) and -gonadal (HPG) axes appear to be modulated by environmentally challenging situations, including neonatal maternal separation or pubertal isolation, it is not surprising that EE can elicit long-lasting changes in mammary ductal development (Figure 8) [10], [27], [28].

This study shows that EE housing leads to a significant reduction of mammary tumor growth as observed previously with models of B16 melanoma, MC38 and APC^min/+^ spontaneous colon cancers [8]. The authors linked the relative tumor resistance in EE mice to a significant decrease in the levels of leptin and increase in those of plasma adiponectin levels. Also, the adiponectin to leptin ratio is higher in individuals with a normal BMI than in overweight or obese individuals [31], [32]. Since leptin promotes breast tumor growth and adiponectin has anticancer properties, it has been suggested that the adiponectin:leptin ratio is a critical factor in mammary cancer tumorigenesis [33], [34], [35]. Our EE housing conditions reduced adiposity and were accompanied with a significant increase in the plasma levels of adiponectin without affecting plasma leptin levels. The increase in the adiponectin:leptin ratio and thus, the balance between these adipokines in EE mice may have limited cancer cell growth in this group (Figure 6). Adiponectin:leptin ratio is significantly increased in EE non-tumor and tumor-bearing mice compared to SE non-tumor and tumor-bearing mice respectively. These results are consistent with previous observations *in vitro* that an increase in the adiponectin:leptin ratio reduces MCF-7 and T47-D cells proliferation [36].

Female C57BL/6 mice have higher levels of leptin than do male mice [37]. While Cao et al. reported a significant decrease in plasma leptin levels in C57BL/6 male mice subjected to EE, we observed such significant change in C57BL/6 female mice [8]. This discrepancy suggests a gender-specific effect of housing conditions on plasma leptin levels as observed previously on certain affective behaviors in C57BL/6J mice [11]. Nevertheless, plasma leptin levels are significantly reduced in EE tumor-bearing mice even if we were unable to explain this down-regulation or exactly when it occurred between tumor cell administration and animal sacrifice. Perhaps this decrease may have maintained a balance between serum adiponectin and leptin levels, and possibly contributed to the tumor inhibition observed in EE mice.

Animals housed in a standard laboratory conditions are relatively overweight, insulin resistant and hypertensive, and are likely to experience premature death [38]. The fact of reducing total daily food intake has been shown to increase the adiponectin:leptin ratio, and it significantly reduces the risk of developing cancer [39], [40]. The increase in mammary tumor growth observed in the SE animals might well reflect similar characteristics observed in obese mice or those fed a high-caloric diet, and is consistent with the long-established relationship between obesity and tumor growth. Indeed, in a study with the same syngeneic breast cancer model, but using ovariectomized C57BL/6 female mice subjected to diet-induced obesity, tumors weighed 233% greater than those of mice on a normal diet [16]. Moreover, although obesity is characterized by a state of chronic low-grade inflammation that may contribute to cancer development and progression. Additionally, reduced adiponectin:leptin ratio associated with obesity, provides a permissive environment for tumor development. However, levels of systemic inflammation markers did not show any significant differences in SE and EE non-tumor-bearing mice. Both SE and EE tumor-bearing mice presented a significant increase in systemic inflammatory markers, including, IL-6, TNF-α, MCP-1, PAI-1 and resistin very likely due to the presence of the tumor. Since TNF-α has been reported to inhibit adiponectin expression, its increase could be responsible for the significant decrease in adiponectin observed in SE and EE tumor-bearing mice but not in non-tumor-bearing mice [41], [42].

Tumor growth is influenced by the tissue microenvironment, which is likely influenced by various systemically affecting factors, including inflammation. Inflammation is known to cause cancer initiation, promotion, angiogenesis and metastasis. Obesity was associated with inflammation in the mouse mammary gland and was accompanied by elevated levels of TNF-α, IL-1β, and COX-2 in both the mammary gland and visceral fat [22]. Similarly, the increase in COX-2 expressing cells in the normal mammary gland of SE mice, suggests that standard housing conditions may trigger an inflammatory state. In these conditions, COX-1 and COX-2 synthesize inflammatory bioactive prostaglandins from endogenous arachidonic acid [43]. COX-2 can be induced by pro-inflammatory and mitogenic stimuli in tissue, and is generally overexpressed in human solid tumors, including 40% of breast cancers and is associated with increased proliferation, high histological grade, metastasis and reduced survival [13], [44], [45]. Furthermore, while COX-2 overexpression is effective in inducing mammary gland tumorigenesis, treatment with COX-2 inhibitors or gene ablation may reduce experimentally induced breast cancers [44], [46], [47]. Tumors in SE mice expressed higher levels of COX-2 (Figure 5), which is consistent with significantly increased cell proliferation marker, Ki67 (Figure 3). Interestingly, tumors expressing the highest levels of COX-2, particularly those isolated from SE mice presented elevated intratumoral leptin levels (Figure 5C and 5D). In addition, high leptin expression in human breast cancer was significantly associated with high Ki67 expression [48]. As observed previously with macrophage, adenocarcinoma cell lines and human umbilical vein ECs (HUVEC), intratumoral leptin expression may promote COX-2 expression in EO771 mammary tumor cells, and subsequently, cell proliferation [49], [50], [51].

Finally, the role of free radicals is important in carcinogenesis. F_2_-isoprostanes, prostaglandin-like novel bioactive compounds are formed during free-radical catalyzed peroxidation of arachidonic acid, and are now regarded as one of the most reliable indicators of oxidative stress *in* *vivo* [52], [53]. In addition, the role of PGF_2α_ in inflammation is well documented [43]. We observed that both PGF_2α_ metabolite and F_2_-isoprostanes are detected immunohistochemically in the tumor isolated either from both SE and EE housed mice (Figure S1). However, urinary levels of these compounds were not statistically significant between the groups. This is possibly due to the fact that high individual variation in these parameters in urine among mice exists and also to the fewer numbers of samples in each category due to sample pooling from several mice of the same group for analysis. However, the existence of both PGF_2α_ metabolite and F_2_-isoprostanes was seen in the microenvironment. Together, this study shows that both PGF_2α_ and F_2_-isoprostanes are found locally in the tumor but with no overall significant difference between groups, which indicates that both free radicals and COX are well involved locally in tumor development and their systemic levels need to be further evaluated in studies with more mice in each group.

Thus, EE conditions induce precocious mammary gland development and significantly decreases mammary tumor growth in experimental mice than do standard laboratory conditions. EE housing also increases serum adiponectin levels without affecting systemic oxidative stress and inflammatory markers such as F_2_-isoprostanes, PGF_2α_, leptin, IL-6, MCP-1, PAI-1, resistin or TNF-α. The imbalance between adiponectin and leptin in SE mice may be one of the factors contributing to tumor aggressiveness. It may lead to increased intratumoral inflammation and angiogenesis and consequently to more aggressive cancers.

In addition, since standard housing affects basic biological processes, such as mammary gland development and disease pathogenesis, EE housing should be used as a potential alternative to work on healthy animals, particularly in studies using mouse mammary tumor models. Finally, this model provides good evidence supporting the positive impacts of physical and social well-being.

This is the first study to show that an enriched environment housing influences mammary gland development in mice and also has an impact on COX-2 and adipokines in mammary tumor growth. COX-2 and a balance in adipokines may have a crucial roll in breast cancer. This study provides further evidence for the need of strong recommendations for future directives on the use of enriched environment in experimental animal models.

## Materials and Methods

This study was conducted in accordance with ethical guidelines [54] and with approval from the Local Ethics Committee (Université d’Auvergne). Female 3-week-old C57/BL6 mice purchased from Charles River Laboratories (Lyon, France), were housed in a temperature- and humidity-controlled room and maintained on a 12 h light/dark cycle and had *ad libitum* access to food and water.

### Environmental Enrichment and Standard Laboratory Housing Conditions

The mice (n = 60) were randomly assigned either to standard laboratory conditions (standard environment: SE) (25 × 20 × 15 cm; 5 mice per cage) or to enriched environment (EE) cages (60×38×20 cm; 10 mice per cage) supplemented with a running wheel, tunnels, igloos, nesting material and wooden toys that were changed once a week for new toys of different shapes and colors (Figure 1B and video S1) [55]. Both type of cages were kept in the same room with a distance apart. Nine weeks after EE or SE housing, 7 mice per group were subjected to body composition analysis. The rest of the mice from both group were subjected to the tumor (n = 14) or vehicle (n = 8) implantation and kept in their respective environment for twenty one more days until sacrifice.

### Mammary Adenocarcinoma Cell Line

The syngeneic EO771 spontaneous mammary adenocarcinoma cell line (mouse ER+ breast cancer) was kindly provided by Dr. Mikhail G. Kolonin at the University of Texas Health Science Center, Texas, USA [15], [16]. The cells were maintained in complete RPMI 1640 Medium (Biowest, Nouaille, France) supplemented with 10% fetal calf serum (Biowest), 100 µg/mL streptomycin (Sigma-Aldrich, Lyon, France), 100 U/mL penicillin (Sigma-Aldrich), 2 mM glutamine (Sigma-Aldrich) and cultured at 37°C in a 5% CO2 humidified atmosphere. Prior to injection, EO771 cells (approximately 80% confluence) were trypsin detached, filtered to prevent cell clumping, mixed with growth factor–reduced Matrigel™ Matrix (BD Matrigel™ Matrix, BD Biosciences, Bedford, MA) and kept on ice until administration to mice.

### Tumor Implantation

3-week-old C57BL/6 mice were randomized to live in either EE (ten mice per cage) or in SE (five mice per cage) for 9 weeks (Figure 1A). Mammary cell line EO771 (5×10^5^ cells in 100 µl) or vehicle only (Growth Factor Reduced BD Matrigel™ Matrix) were orthotopically transplanted into the fourth right mammary fat pad of both EE and SE mice. From day 6 after tumor cells injection, tumor size was measured three times per week and tumor volume was calculated according to the formula V = 0.52× width^2^ × length, where width is the smaller of the two measurements. Twenty-one days after the cell injections, the mice were anesthetized with ketamine/xylazine (i.p., 100/10 mg/kg, Sigma-Aldrich), and blood samples were collected by cardiac puncture. After blood collection, the tumor and major organs were harvested. The tumors were stored frozen in liquid nitrogen for protein or embedded in Optimal Cutting Temperature compound (Tissue-Tek®, Sakura Finetek USA, Torrance, CA) for immunofluorescence study or formalin-fixed and paraffin-embedded for routine histological examinations with H&E staining.

### Body Composition Analysis

Nine weeks after EE or SE housing, 7 mice per group were subjected to magnetic resonance imaging (MRI) using Echo MRI (Echo Medical Systems, Houston, TX) to determine the body composition. Following MRI analysis (n = 7), the mice were sacrificed and blood was collected and other organs (mammary fat pad, visceral fat, kidneys, liver, lungs, spleen and brain) were excised and weighed. The different skeletal muscles from both hind legs, tibialis anterior, plantaris, gastrocnemius, soleus and exterior digitorum longus were dissected and pair-weighed.

### Antibodies Applied in Immunohistochemistry

The rabbit antibodies used in the study at the dilutions stated were as follows: rabbit monoclonal antibody to COX-2 was purchased from Abcam (ab21704, Abcam, Cambridge, UK). The anti-8-iso-prostaglandin-F_2α_ (1∶1000) and anti-15-keto-13,14-dihydro-prostaglandin F_2α_ (1∶1000) were raised in rabbits as previously described [56], [57], [58], [59], [60]. The anti-cleaved caspase 3 (1∶1000; 9661) was purchased from Cell Signaling, the anti-PECAM-1 (CD31; 1∶150; SAB4502167) from Sigma-Aldrich, the anti-mKi67 (1∶100; AB9260) from Millipore (Millipore, Molsheim, France), the anti-K14 from Covance Research Products (1∶5000; PRB-155P) and the anti-actin from Abcam (1∶1000; Ab1801). Goat antibodies specific for mouse leptin (AF498), ObR (AF497), and adiponectin (AF1119), were from R&D Systems (R&D Systems, Lille, France) and were used at 5 µg/ml. The goat anti-COX-2 (4 µg/ml; Ab23672) was purchased from Abcam.

For indirect immunofluorescence staining, AlexaFluor 488 or 555–conjugated donkey anti–rabbit or anti–goat IgG (Invitrogen, Paisley, UK) were diluted at 1∶1000 and used for detection of primary antibodies. For immunoblotting, horseradish peroxidase-conjugated donkey anti–goat (Abcam) or anti–rabbit IgG (Thermo Fisher Scientific, Illkirch, France) were used at 1∶5000 and 1∶1000, respectively.

### Histology, Immunofluorescence Staining and Confocal Microscopy

Unless otherwise mentioned, all chemicals were purchased from Sigma-Aldrich, (Lyon, France). Various tissues were fixed in Excell Plus (Microm Microtech France), embedded in paraffin, cut at a thickness of 5 µm and stained using H&E. For frozen sections, tissues were embedded in optimal cutting temperature compound. Sections (10 µm), were stained by indirect immunofluorescence as previously described (30), counterstained with 4′,6-diamidino-2-phenylindole (0.5 µg/ml) and mounted in a drop of Mowiol (Calbiochem, France). For negative controls, sections were incubated without primary antibodies. Confocal laser-scanning microscopy was performed with the Leica TCS SP5 MP confocal and multiphoton microscope system (Leica Inc., Heidelberg, Germany). Digital images were prepared using the Leica LAS-AF Lite Software, and were further processed using Adobe Photoshop CS5.1 and compiled using Adobe Illustrator CS5.1 (Adobe Systems Inc., San Jose, CA). The average number of stained positive cells or positive area per microscopic field (magnification 20X) was quantified using the particle analyzer from ImageJ software (National Institutes of Health, Bethesda, MD). Images were subjected to the threshold function using the same threshold for all images for a group and then the number of particles in the image was then counted. Particles less than 30 pixels were excluded. Each quantification corresponds to the average number of positive cells observed in at least six different fields. Adipocyte size quantification was done using ImageJ software [61].

### Western Blotting of Tissue Extract

Collected tumors were homogenized with an Ultra-Turrax T25 (Ika Labortechnik, Staufen, Germany) in radioimmunoprecipitation assay lysis buffer (Sigma-Aldrich) containing protease inhibitor cocktail (P8340, 1∶100; Sigma-Aldrich) and phosphatase inhibitor cocktail (P5726, 1∶100; Sigma-Aldrich). The homogenates were centrifuged for 15 min at 15,000 rpm, and the supernatants were kept at −70°C until used. Protein concentrations were measured using the bicinchoninic acid (BCA) protein assay (Thermo Fisher Scientific) and equal amounts of protein were resolved by 10% SDS-PAGE before transfer to Hybond nitrocellulose membrane (GE Healthcare, Buckinghamshire, UK). Membranes were probed with primary and HRP-conjugated secondary antibodies and visualized with ECL (GE Healthcare) [62]. The relative abundance of each band was quantified by using ImageJ. COX-2 and leptin expression were normalized to β-actin before the percentage relative to control was calculated.

### Whole-mount Staining of Mammary Glands

Whole-mount of mammary glands was performed as described previously [63]. Briefly, inguinal (no. 4) mammary glands were dissected from mice fixed overnight in Carnoy′s solution (60% ethanol, 30% chloroform and 10% glacial acetic acid), hydrated, stained overnight with carmine alum (0.2% carmine, 0.5% aluminum potassium sulfate), dehydrated in graded solutions of ethanol, cleared in xylene substitute and mounted. Epithelial growth, primary branching and lateral side branching were assessed in whole mounts of the fourth inguinal mammary gland as previously described [64]. Epithelial growth corresponds to the distance from the lymph node to the end of the epithelium. Primary branches were defined as ducts extending from the nipple and terminating in an end bud. Primary branching and side-branching were evaluated independently in a blind fashion by two persons with a Motic 45° Binocular head by counting the number of branch points along the terminal ductal tips. Results were expressed as the ratio of branch points per duct.

### Metabolic, Oxidative and Inflammatory Markers Quantification

Urinary concentrations of 8-iso-PGF_2α_, a major F_2_-isoprostane and a currently the most reliable biomarker of *in vivo* oxidative stress, were determined using a radioimmunoassay as previously described [57]. Urinary concentrations of 15-keto-dihydro-PGF_2α_, a major metabolite of arachidonic acid-derived (through COX) primary PGF_2α,_ were determined using a radioimmunoassay as previously described [56]. Concentrations of both 8-iso-PGF_2α_ and 15-keto-dihydro-PGF_2α_ were adjusted for urinary creatinine concentrations measured by a Kone Lab 20 (Thermo Clinical Lab Systems, Vantaa, Finland). Plasma adiponectin levels were measured using ELISA according to the manufacturer’s instructions (Mouse Adiponectin ELISA Kit, Millipore, France). Plasma concentrations of interleukin (IL)-6, leptin, TNF-α, resistin, PAI-1, and MCP-1 were measured simultaneously using a Luminex xMAP system (MILLIPLEX MAP Mouse Adipokine Panel, Millipore, France). The mean fluorescence intensity (MFI) was detected by the Multiplex plate reader for all measurements (Luminex System, Bio-Rad Laboratories, Germany).

### Cell Proliferation Assay

EO771 cells were seeded in a 96-well plate at 8000 cells per well in complete RPMI 1640 Medium and were allowed to adhere for 24 h. The following day, cells were shifted to serum-free medium for 20 hours and then treated with RPMI1640 medium plus recombinant mouse leptin (0, 10, 25, 50, 100 or 200 ng/ml) (R&D Systems) or with RPMI1640 medium plus serum samples from individual mice collected at the time of sacrifice (used for body composition analysis, 9 weeks EE or SE, 12 weeks of age) (1% of mouse serum, n = 7 in each group). Serum were pre-incubated or not with leptin neutralizing antibody at 4°C for 12 h (0.05 µg/ml, AF498, R&D Systems). After 24 h of treatment, supernatant fractions were removed and proliferation assay was performed using the resazurin reduction test [65]. Cells were incubated for 2 h with a solution of resazurin diluted in RPMI1640 medium (25 µg/ml, Sigma-Aldrich). Fluorescence was measured by a Fluoroskan Ascent Fl™ plate reader (Labsystem SA, Les Ulis, France).

### Statistical Analysis

The data were expressed as the mean ± SEM. Statistical calculations were performed using StatView® 5.0 statistical software for Windows (SAS Institute Inc., USA). Differences between the two groups were assessed using the nonparametric Mann-Whitney test. When four groups were compared, statistical analysis was performed by two-way multivariate analysis of variance (ANOVA) followed by a Fisher’s PLSD post hoc test (StatView®). Statistical significance was assumed if p<0.05.

## Supporting Information

Figure S1 **Analysis of 8-iso-PGF_2α_ and PGF_2α_ metabolite by indirect immunofluorescence staining in tumors from 12-week-old mice housed in SE and EE cages for 9 weeks.** (A) Tumor sections were labeled by indirect immunofluorescence staining for 8-iso-PGF_2α_ antibody (green) and with DAPI as nuclear counterstain (blue). (B) Tumor sections were labeled by indirect immunofluorescence staining for 15-keto-dihydro-PGF_2α_ antibody (green) and with DAPI as nuclear counterstain (blue). Right panels in A and B: Immunopositive stained area were measure using ImageJ software (n = 3 per group). The control levels were set at 100%. Upper panels show area with low detection. Lower panel shows area with high detection. Scale bars: 100 µm. Data presented are the mean ± SEM; NS = not significant.
(TIFF)

Video S1 **EE housing conditions stimulate physical and social activity compared to SE conditions.** EE increases social stimulation through increased number of animals per cage. In this larger environment, mice explore, climb, and run on exercise wheel. Moreover, EE contains nesting, hiding places and *ad libitum* access to food.
(WMV)
