# Supplementary figures and images for: Effects of Enriched Environment on COX-2, Leptin and Eicosanoids in a Mouse Model of Breast Cancer
Source: PLoS One. 2012 Dec 13;7(12):e51525. doi: 10.1371/journal.pone.0051525 (PMC3521763; doi:10.1371/journal.pone.0051525)

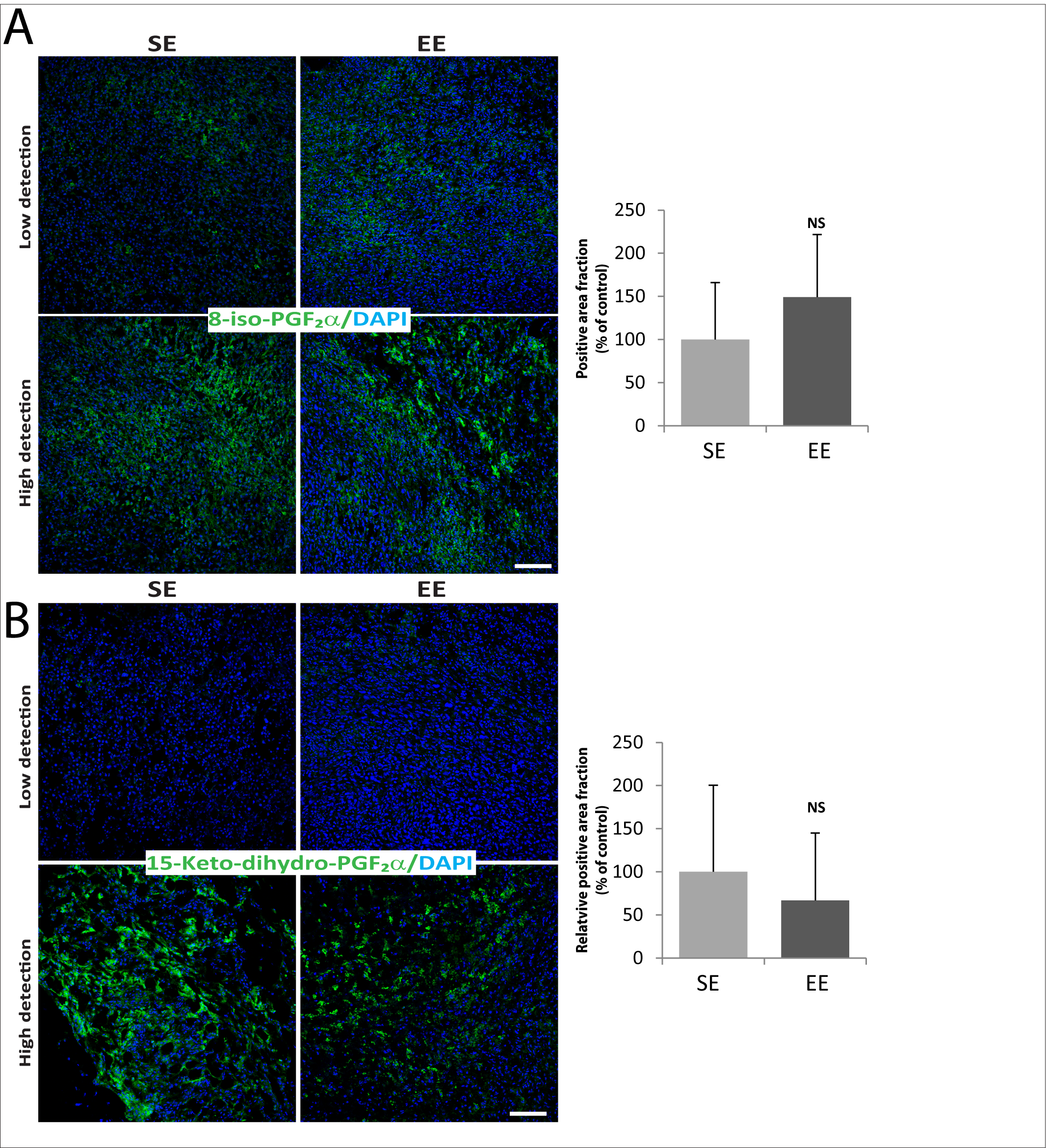

Supplement: Figure S1 — Analysis of 8-iso-PGF2α and PGF2α metabolite by indirect immunofluorescence staining in tumors from 12-week-old mice housed in SE and EE cages for 9 weeks. (A) Tumor sections were labeled by indirect immunofluorescence staining for 8-iso-PGF2α antibody (green) and with DAPI as nuclear counterstain (blue). (B) Tumor sections were labeled by indirect immunofluorescence staining for 15-keto-dihydro-PGF2α antibody (green) and with DAPI as nuclear counterstain (blue). Right panels in A and B: Immunopositive stained area were measure using ImageJ software (n = 3 per group). The control levels were set at 100%. Upper panels show area with low detection. Lower panel shows area with high detection. Scale bars: 100 µm. Data presented are the mean ± SEM; NS = not significant. (TIFF) [file pone.0051525.s001.tiff]
